# Rooted Soil Shear Apparatus: A low-cost, direct shear apparatus for measuring the influence of plant roots on soil shear strength

**DOI:** 10.1016/j.ohx.2025.e00726

**Published:** 2025-11-29

**Authors:** Gianmario Sorrentino, Gerald Innocent Otim, Alena Zhelezova, Irene Rocchi

**Affiliations:** Technical University of Denmark (DTU), Department of Environmental and Resource Engineering, 2800 Kgs. Lyngby, Denmark

**Keywords:** Root-reinforced soil, Direct shear test, Arduino, Raspberry Pi, Soil stabilization, Bio-based ground improvement

## Abstract

Rooted Soil Shear Apparatus (RSSA) is an open-source laboratory apparatus designed to quantify the effect of plant roots on soil shear strength. Traditional methods used to assess the effect of vegetation on soil strength often rely on expensive proprietary systems and can involve sample disturbance, which may alter the root-soil interactions. This novel apparatus offers both an Arduino-based and a Raspberry Pi solution for data acquisition and control. The device enables laboratory shear testing directly in the same polyvinyl chloride (PVC) pots where the plants grow, eliminating the need to disturb the root-soil structure. Validation experiments demonstrate its effectiveness in capturing shear strength variations in rooted and non-rooted soil samples. By providing an affordable and customizable alternative to conventional shear testing equipment, the RSSA device advances research in geotechnical engineering and soil stabilization.

## Graphical abstract

**Figure:**
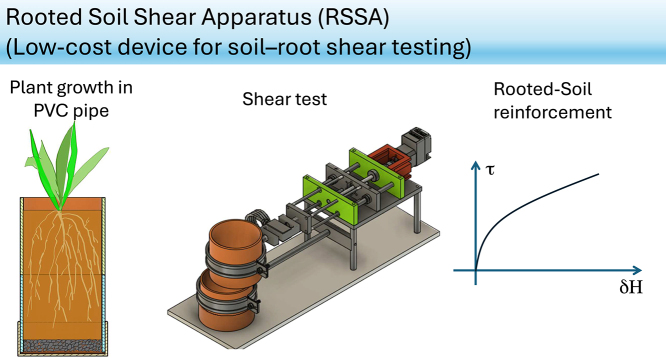

**Specifications table**

**Table:** 

Hardware name	Rooted soil shear apparatus

Subject area	Engineering and material science

Hardware type	• *Measuring physical properties and in-lab sensors* • *Mechanical engineering and materials science*

Closest commercial analog	No commercial analog is available

Open source license	CC BY-NC

Cost of hardware	€1500

Source file repository	doi:10.17605/OSF.IO/W8DB5 (OSF: https://osf.io/w8db5/)

	

## 1 Hardware in context

**Fig. 1:**
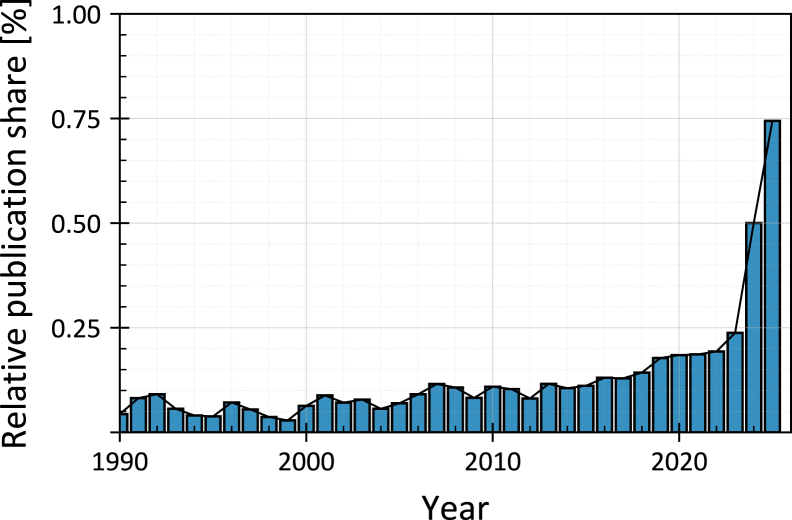
Relative proportion of publications on rooted-soil strength within civil engineering (normalized to total civil engineering publications per year). Data source: [4], search terms: “rooted-soil strength”, filtered to civil engineering articles.

Roots play a critical role in stabilizing slopes and preventing erosion, a beneficial effect that has been well-documented in the literature [1], [2], [3]. Scientific interest in the role of plant roots in enhancing soil strength has increased in recent years within civil engineering research, as shown in Fig. 1.

However, accurately quantifying the contribution of plant roots to soil shear strength remains a significant challenge. Currently, there is no standardized testing equipment specifically designed to measure this reinforcement effect. Consequently, researchers have developed their customized experimental setups, often inspired by classical geotechnical testing apparatus, to assess rooted soil strength. Among these, the direct shear apparatus [5] is the most commonly used.

Laboratory direct shear tests are widely used to assess how plant roots influence soil shear strength by comparing rooted soil to barren soil. This type of equipment is frequently reproduced due to its relative simplicity. In this setup, the soil block is sheared horizontally, typically under its self-weight or an applied normal load. Some researchers use traditional direct shear devices and insert rooted soil samples [6], [7], [8], [9], but these setups are often limited by small sample sizes–particularly in height–such as the standard 20 mm blocks commonly used in geotechnical soil testing [5]. Many researchers have developed custom testing setups to overcome the limitations of traditional direct shear devices [10], [11], [12]. Some studies suggest that investigating root effects may require larger samples (e.g., 300 × 300 × 200 mm) to better capture root-soil interactions [2], [13]. However, large-scale direct shear apparatuses tend to be highly expensive and difficult to accommodate in standard laboratory environments. Moreover, many of these custom-built setups lack clear technical specifications, making reproducibility across research groups a significant challenge.

A promising approach to improving the accessibility and reproducibility of laboratory testing is the integration of microcontrollers and single-board computers, such as Arduino and Raspberry Pi. These technologies offer cost-effective, highly customizable platforms for experimental setups [14], [15], [16]. Their widespread availability and strong support communities enable researchers to develop scalable and reproducible testing apparatuses without the high costs associated with proprietary equipment. Additionally, the use of open-source hardware and software makes it significantly easier to replicate experimental setups, exchange code, and collaboratively refine testing protocols, promoting a more transparent and efficient research environment.

Building on these advancements, we present a novel direct shear device that incorporates Arduino and Raspberry Pi for control and data acquisition. A key feature of our design is the ability to shear polyvinyl chloride (PVC) pots in which plants grow directly, eliminating sample disturbance during preparation, setup, and testing. This preserves undisturbed root-soil interactions, allowing for a more realistic representation of natural conditions. Additionally, our apparatus is economically viable and suitable for large-scale investigations, making advanced soil-root interaction studies more accessible to researchers across disciplines.

In summary, the development of standardized, reproducible, and cost-effective testing equipment is essential for advancing the understanding of root-soil interactions and their impact on soil shear strength. Taking advantage of modern microcontroller and computing technologies, our apparatus provides a robust solution that overcomes the limitations of existing bespoke setups, facilitating more consistent and scalable research in this critical area of geotechnical engineering.

## 2 Hardware description

**Fig. 2:**
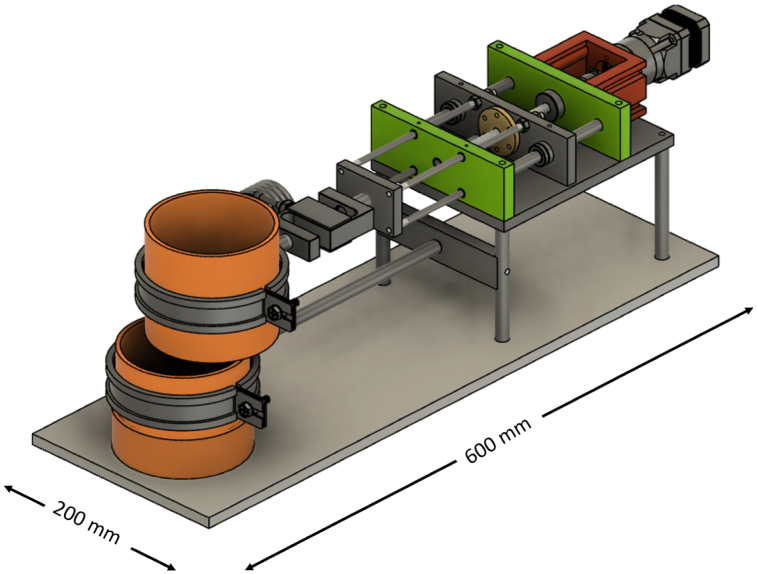
Schematic of the Rooted Soil Shear Apparatus device.

The shear apparatus developed is designed to quantify the influence of plant roots on the shear strength of soil in pot experiments, a parameter of significant importance in geotechnical engineering and soil science. The apparatus is compact and laboratory-friendly, featuring a base plate measuring 200 mm in width and 600 mm in length, allowing it to be conveniently placed on standard laboratory benches. The apparatus is shown in Fig. 2.

The Rooted Soil Shear Apparatus (RSSA) device is made of aluminium, chosen for its affordability, durability, and lightweight properties, which improve portability and ease of handling while maintaining stability during testing. The frame is securely screwed to the base plate, providing a stable foundation, and supports the linear actuator responsible for generating the controlled horizontal displacement during shear tests.

**Fig. 3:**
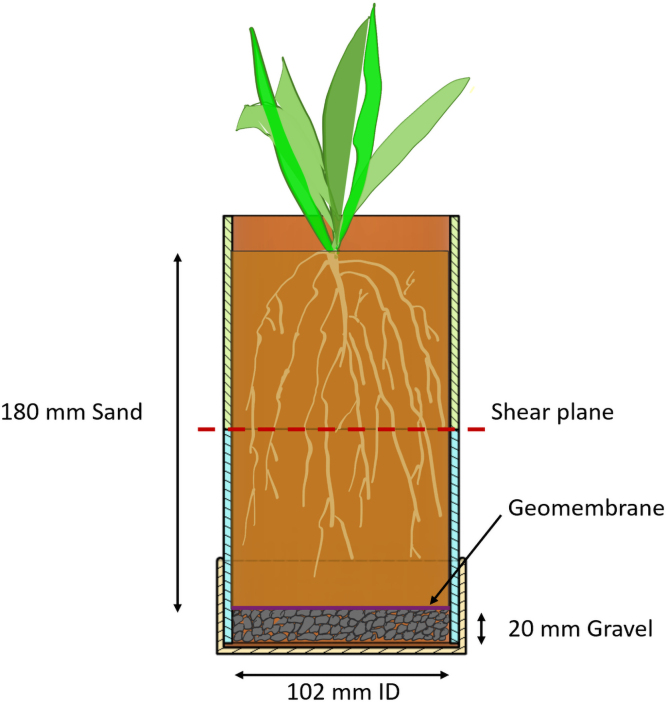
Schematic of the PVC pipe utilized for plant root growth and as the shear chamber in the experimental setup.

PVC pipes are utilized both as pots for plant growth, creating rooted soil specimens (Fig. 3), and as shear chambers. The PVC pipes used as sample holders have an external diameter of 110 mm, an internal diameter of 100 mm, and a wall thickness of approximately 5 mm. Using PVC pipes as pots for plant growth to assess the effects of roots on soil shear strength is a well-established approach in the literature [6], [8], [11], [17].

This integrated design prevents movement of the soil and plant during testing, thereby enhancing the quality and consistency of the samples.

At this scale, the setup is appropriate for herbaceous plants commonly used for shallow slope stabilization (e.g., vetiver, clover, switchgrass) [18], [19] and for seedlings of shrubs or trees (e.g., poplar, willow) during their first year of growth [20], when root systems remain confined within small pots. The apparatus could also be used to test samples prepared directly inside pipes for bio-based stabilization methods such as microbially induced calcite precipitation (MICP) [21], [22], [23] or fungal mycelium treatments [24], [25], as well as biopolymer-treated soils [22], provided the material remains weakly to moderately bonded (i.e., poorly cemented), so that shearing can be carried out within the targeted stress range of the current setup. Larger vegetation with more extensive root systems, or strongly cemented/high-strength materials, would require scaling the apparatus and/or upgrading the load cell and drive system; such developments are identified as future work.

The PVC pipe is cut into two parts across the pre-determined shear plane and secured together with tape during the plant growth phase to avoid soil falling out. Before testing, the complete pot-including the plant and soil-is positioned within the shear device. The plate of the shear box features a custom slot to accommodate the pot, with the lower half fixed securely to a pipe clamp attached to a stationary arm connected to the frame. This ensures that the lower portion of the sample is fixed throughout the test.

Conversely, the upper half of the pot is secured to a moving arm, which is connected to a lead screw driven by a Nema 17 stepper motor. This configuration converts the rotary motion of the motor into precise linear displacement, enabling controlled horizontal movement of the upper block while the lower block remains fixed.

An S-type load cell, with a maximum force capacity of 300 kg and an accuracy of ±10 g, is installed between the lead screw and the pipe clamp holding the upper half of the PVC pipe to measure applied shear and horizontal forces accurately. Additionally, a linear displacement sensor monitors the horizontal displacement of the upper block of the specimen.

Fabrication of the shear apparatus was carried out at the DTU (Technical University of Denmark) workshop using standard tools and machinery commonly found in university workshop spaces. Computer numerical control (CNC) milling machine was employed due to its availability in our facility; however, the equipment can also be fabricated without the use of CNC machining.

Both Arduino and Raspberry Pi offer viable solutions for data acquisition and control in laboratory experiments, each with distinct advantages. The Arduino interfaces with the user through Excel Data Streamer, providing a straightforward and efficient method for data logging and real-time monitoring. In contrast, the Raspberry Pi features its own graphical user interface (GUI), offering a more integrated and flexible platform for experiment control and data processing. This flexibility allows researchers to choose the most suitable system based on their specific testing requirements.

In summary, the proposed device can support research on bio-based ground improvement and rooted-soil mechanics by:

• Allowing comparative shear tests in growth pots–rooted soil vs. root-free soil–to isolate root effects with minimal disturbance.
• Providing a low-cost, open-source system (Arduino/Raspberry Pi) for force–displacement data acquisition and easy code modification.
• Supporting bio-based stabilization studies by shear-testing specimens directly in their curing pipes.
• Providing a robust, compact rig with Arduino/Raspberry Pi instrumentation, well-suited to teaching labs where students gain hands-on hardware and coding experience.

### 2.1 Control and acquisition system

A direct shear test typically involves acquiring data on displacement and shear forces in both horizontal and vertical directions, and controlling horizontal displacement to be constant. In this setup, only the horizontal load and horizontal displacement are measured, as the confinement stress in the soil is maintained by the suction due to partial saturation conditions. In the current configuration, no external normal load is applied and tests are therefore conducted under self-weight only. Matric suction is not actively controlled in the current configuration, but given the influence of suction in this context, incorporating a tensiometer could provide valuable insights into pore-water pressure and improve understanding of soil behavior during testing. An example of a suitable instrument is the TEROS 31 tensiometer (Meter Group, USA) [26], which has a shaft diameter of 5 mm and is available in lengths from 2 cm to 20 cm. This tensiometer can be inserted directly into the soil like a pin, typically about 10 mm above the shear plane, to record suction close to the zone of shearing with minimal disturbance.

During testing, it is essential to control the imposed shear rate, which is achieved using the stepper motor. Consequently, the system must be capable of reading an analog signal from a Linear Variable Differential Transformer (LVDT), a differential signal from the load cell, and controlling the stepper motor.

Arduino and Raspberry Pi were chosen for this task as either option provides a cost-effective and reliable solution. Both offer flexibility, and their use allows for a comparison of their performance in the setup, which also facilitates the way for future developments and improvements.

#### 2.1.1 Arduino-based control and acquisition system

**Fig. 4:**
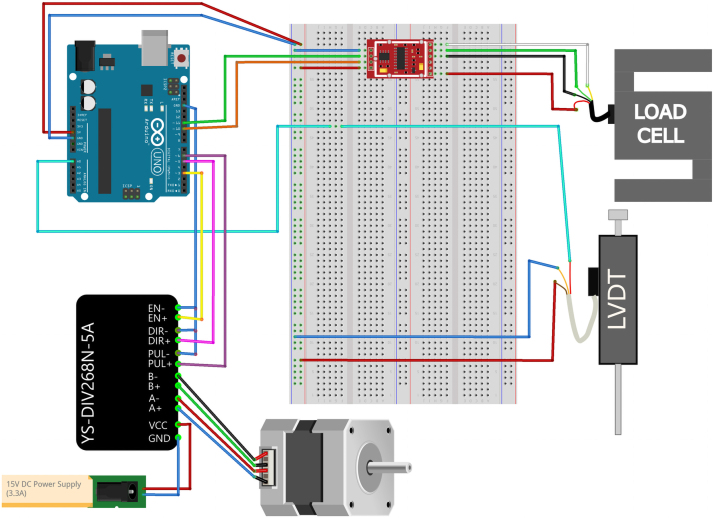
Schematic representation of the Arduino-based wiring for the RSSA device.

Fig. 4 shows the schematic of the Arduino-based control and acquisition system.

The Arduino is responsible for reading the signals from the load cell and the LVDT. The LVDT provides a single-ended analog voltage output signal, which is read through the analog input pins of the Arduino. On the other hand, the load cell outputs a differential signal, which the Arduino cannot read directly, as it only accepts single-ended signals. To address this, an amplifier (HX711) is used to convert and amplify the differential signal from the load cell into a single-ended signal that can be processed by the Arduino unit. The HX711 library is used to interface with the amplifier, ensuring an efficient data acquisition.

To control the stepper motor, the YS-DIV268N-5 A motor driver was selected for its ability to provide smooth and precise control of the stepper motor. This driver is compatible with both Arduino and Raspberry Pi platforms, making it a versatile and easy-to-implement solution for controlling the stepper motor within the system.

The Arduino sketch is designed to monitor sensor data and control the stepper motor for the shear test.

The stepper motor driver is controlled by three pins: one for the step signal, one for the direction, and one to enable or disable the motor. The motor’s movement is governed by the period specified by the user, allowing for precise control over the shear rate. The system accepts commands via the serial interface, enabling the user to adjust settings during the test. The available commands include setting the step delay period (p<number>), moving the motor forward (f), moving it in reverse (r), or stopping the motor immediately (s).

The Arduino sketch implements a timing mechanism to ensure that the sensor readings are updated periodically, with optional individual intervals for the LVDT and load cell. The motor’s stepping is also controlled by time-based intervals, allowing for smooth operation of the motor during testing.

Real-time data, including the LVDT voltage, load cell value, and system time, is continuously output to the serial port and streamed to an Excel interface for both data acquisition and control of the Arduino unit providing the user with feedback on the system’s performance. The Excel Data Streamer serves as a bridge between the Arduino and the user interface, enabling seamless integration of control commands and data collection.

The full Arduino sketch is available on GitHub at https://github.com/GM1710/RSSA, where it can be accessed for further review or modification.

#### 2.1.2 Raspberry pi-based control and acquisition system

**Fig. 5:**
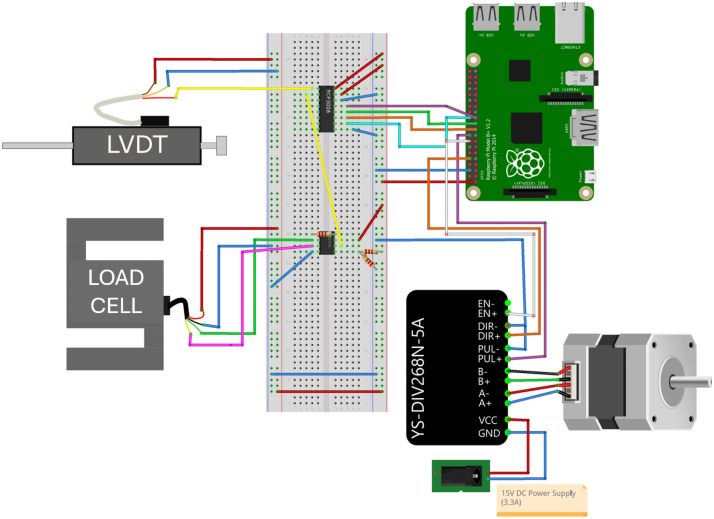
Schematic representation of the Raspberry Pi-based wiring for the RSSA device.

Fig. 5 shows the schematic of the Raspberry Pi-based control and acquisition system.

The Raspberry Pi is connected to an Analog-to-Digital Converter (ADC) via SPI (Serial Peripheral Interface) communication to enable the reading of analog sensor signals, since the Raspberry Pi lacks native analog input pins.

The LVDT outputs an analog voltage signal, which is directly connected to one of the ADC’s input channels. Conversely, the load cell generates a differential signal, which must first be amplified before being processed. For this purpose, an amplifier is used to amplify and convert the differential output of the load cell into a single-ended signal, which is then fed into the ADC.

To control the stepper motor, the Raspberry Pi is directly interfaced with the YS-DIV268N-5 A motor driver. This driver allows precise control of the stepper motor through GPIO signals from the Raspberry Pi, enabling adjustments to the shear rate as required by the testing procedure.

This setup ensures a fully integrated control and data acquisition system, leveraging the computational power of the Raspberry Pi while maintaining compatibility with analog sensor inputs.

The Raspberry Pi software performs the same core functions as the Arduino, enabling sensor monitoring, motor control, and data acquisition for the RSSA device. However, since the Raspberry Pi lacks native analog input capabilities, an MCP3008 ADC is used to read signals from the load cell and the LVDT. The load cell signal is amplified using an AD623 instrumentation amplifier before being processed by the ADC. SPI communication is used to interface the MCP3008 with the Raspberry Pi, following the example code provided by Adafruit [27].

A Python script, built with tkinter, provides a Graphical User Interface (GUI) for user interaction, shown in Fig. 6. The interface consists of two main windows:

• a motor control panel that allows the user to start, stop, adjust speed, and change the direction of the stepper motor, shown in Fig. 6(a).
• a real-time graph displaying sensor readings, providing visual feedback on the shear test, shown in Fig. 6(b).

The motor operates in a separate thread to ensure smooth and responsive GUI performance. Data acquisition is automated, with sensor readings and elapsed time continuously recorded and saved to an output file for further analysis. This system provides an alternative to the Arduino setup, offering an integrated graphical interface and eliminating the need for an external data streaming tool.Fig. 6Graphical User Interface (GUI) of the Raspberry Pi control system: (a) Motor control panel for adjusting motor speed, direction, and starting/stopping the test; (b) Real-time sensor data display.

**(a):**
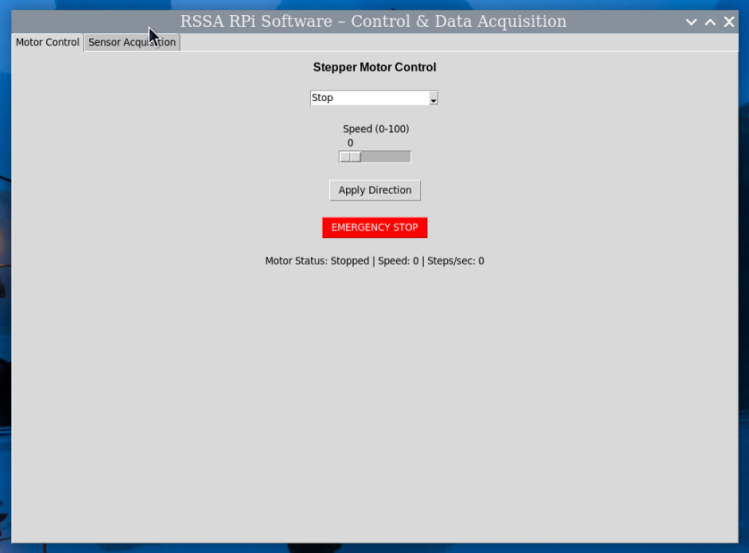
.

**(b):**
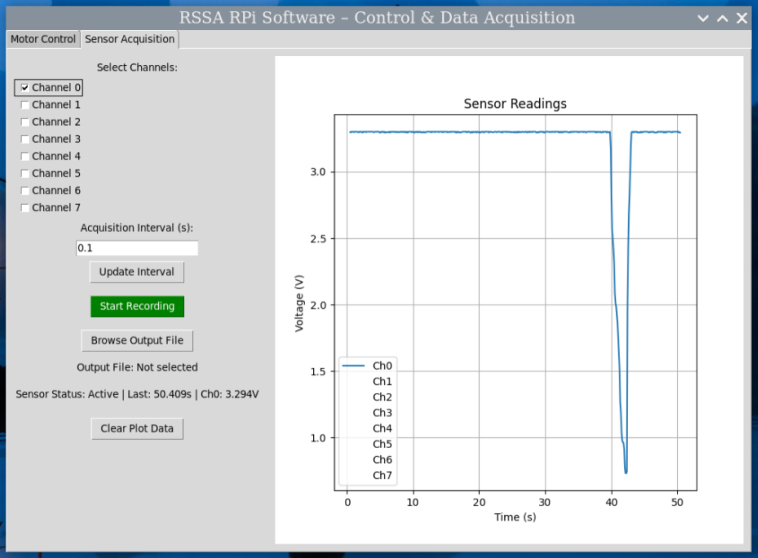
.

The Python script for the Raspberry Pi is available on GitHub at https://github.com/GM1710/RSSA, where it can be accessed for further review or modification.

## 3 Design files

The design of the Rooted Soil Shear Apparatus (RSSA) was realized using Fusion 360. All design files are available in CAD format, and the drawings provided to the workshop for fabrication are accessible in PDF format. Detailed diagrams of the electrical connections and wiring, including all components, are presented in Fig. 4, Fig. 5. Additionally, the software code developed for both the Arduino and Raspberry Pi platforms is available via GitHub repository. Table 1 presents a summary of all design files and resources.

**Table 1:** Summary of design files available in the repository.

Design filename	File type	Location of the file
Rooted Soil Shear Apparatus design	.STEP file	https://osf.io/w8db5/files/osfstorage
Dimensioned Assembly Drawings	PDF	https://osf.io/w8db5/files/osfstorage
Electrical Circuit Diagrams	Image files	Presented in Fig. 4 and Fig. 5 of the article
Software Code for Arduino and Raspberry Pi	Code files	https://github.com/GM1710/RSSA_ShearDevice/tree/main

## 4 Bill of materials summary

The construction of the RSSA requires a comprehensive set of components, which can be categorized into three main parts: the mechanical equipment itself, the Arduino-based setup, and the Raspberry Pi-based setup. Table 2 provides a detailed breakdown of all the materials and components necessary to build and operate the device, including their specifications and the suppliers from which each part was purchased.

**Table 2:** Bill of materials for the rooted soil shear apparatus.

Code	Part	Description	Qty	Unit cost [€]	Total cost [€]	Suppliers
**Rooted Soil Shear Apparatus**						
ASR112	Material	Aluminum rods round 6082 12 mm	1.80 kg	9.33	16.80	LEMVIGH-Müller A/S
AS4360	Material	Aluminum bars square 2007 T4 60 mm	1.00 kg	9.55	9.55	LEMVIGH-Müller A/S
ASF51512	Material	Aluminum rails flat 6060/6063 150 × 12 mm	2.00 kg	7.52	15.04	LEMVIGH-Müller A/S
ASF52001	Material	Aluminum rails flat 6060/6063 200 × 10 mm	5.00 kg	7.64	38.20	LEMVIGH-Müller A/S
ABRI100^a^	Pipe clamps	Lined pipe hangers for threaded rod	2	3.40	6.80	erik-larsen shop
893-7461	Bearing	Deep Groove Ball Bearing	2	5.79	11.58	RS
862-5307	Lead Screw	Lead Screw 10 mm Shaft Diameter	1	64.46	64.46	RS
617-4624	Bearing	Linear Bearing with 19 mm Outside Diameter	2	16.88	33.76	RS
174-6698	Clamp Screw	Ruland Shaft Collar Two Piece	2	16.71	33.42	RS
535-0489	Stepper Motor	Bipolar Stepper Motor Nema-17	1	31.20	31.20	RS
693-2411	Bellows Coupling	Bellows Coupling, 26 mm Outside Diameter, 8 mm Bore, 37.5 mm Length Coupler	1	43.40	43.40	RS
EG17-G10	Gearbox Gear	Gearbox Gear Ratio 10:1 for Nema 17 Stepper Motor	1	40.06	40.06	Stepperonline
172-1273	Bearing	Bearing with 12 mm Outside Diameter	4	12.12	48.48	RS
204-2768	Load Cell	Load Cell, 300 kg Range, Compression, Tension Measure	1	162.22	162.22	RS
TR-0025^a^	Linear Transducer	TR Position Transducer	1	147.42	147.42	novotechnik
521105	PVC Pipe	Sewer pipe 110-1000 mm	2	5.36	10.72	vvs-eksperten
192284000	PVC Pipe Lid	Sewer end sleeve PVC Φ110 mm for bonding	10	10.19	101.88	vvs-eksperten
200-8950	15 Vdc AC/DC-adapter	AC/DC-adapter, 3.36 A , 50.4 W , 2 Pin	1	32.52	32.52	RS
175-2108	Breadboard	Solderless experimental circuit board kit 230 × 175 × 31 mm	1	60.58	60.58	RS
**Arduino**						
791-6463	Jumper Wire	150 mmInsulated Breadboard Jumper Wire	1	3.29	3.29	RS
HX711	Amplifier	Weight Sensor Dual-Channel A/D Module, HX711	1	5.19	5.19	ArduinoTech
715-4081	Arduino	Arduino Uno Rev 3	1	28.97	28.97	RS
**Raspberry Pi**						
182-2096	Raspberry Pi	Raspberry Pi 4 B 4GB	1	60.00	60.00	RS
791-6454	Jumper Wire	150 mmInsulated Breadboard Jumper Wire	1	3.29	3.29	RS
669-6064	MCP3008	ADC MCP3008-I/P, Octal, 10 bit-, 200ksps, PDIP, 16 Pin	1	2.95	2.95	RS
584-AD623ANZ	Amplifier	AD623ANZ	1	9.23	9.23	Mouser Electronics

a Some components were selected based on their availability in the laboratory to optimize cost and accessibility.

## 5 Build instructions

Assembling the RSSA device is a relatively easy procedure, the tools required are an Allen key and a spanner set.

**Fig. 7:**
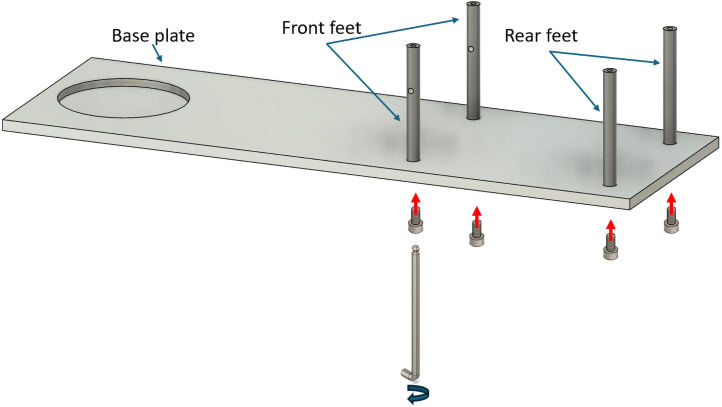
Illustration of the procedure for securing the feet to the base plate, highlighting the correct positioning of the front and rear feet and the use of M6x10 socket screws.

The first step in assembling the RSSA device is to secure the feet of the frame to the base plate, providing a stable foundation for the linear motion assembly that will be installed on top. The feet are attached to the base plate using M6x20 socket screws. There are two types of feet: one pair without threaded holes across the body, which should be installed at the back of the device, and another pair with a threaded hole in the orthogonal direction to their axis, which should be installed at the front. An Allen key should be used to tighten the feet securely to the base plate. This stage of the assembly is shown in Fig. 7.

The assembly of the fixed arm, which holds the bottom part of the pipe, is shown in Fig. 8. Once the fixed arm is assembled (Fig. 8(a)), it should be inserted between the two front feet, as illustrated in Fig. 8(b).

**Fig. 9:**
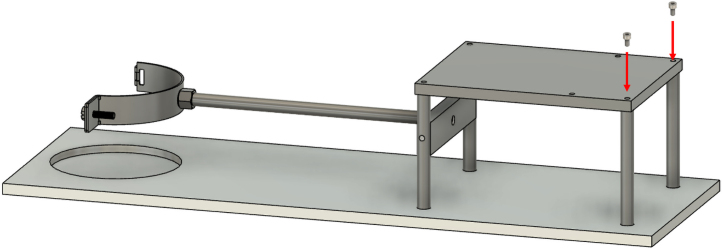
Positioning the top plate of the supporting frame.

Once the fixed arm has been installed, the top plate of the supporting frame can now be positioned. At this stage, only the two back screws are inserted (Fig. 9), and they are left slightly loose to allow for adjustments during the next assembly steps.Fig. 8Assembly of the fixed arm to the RSSA device: (a) assembly of the fixed arm, showing the individual components and their arrangement before installation on the shear device; (b) attachment of the fixed arm to the RSSA device, illustrating the alignment and securing process.

**(a):**
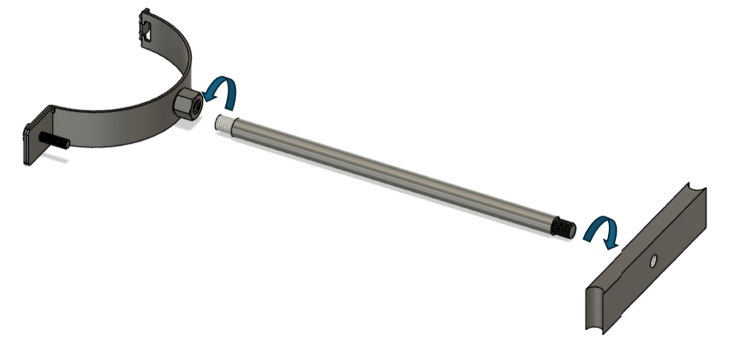
.

**(b):**
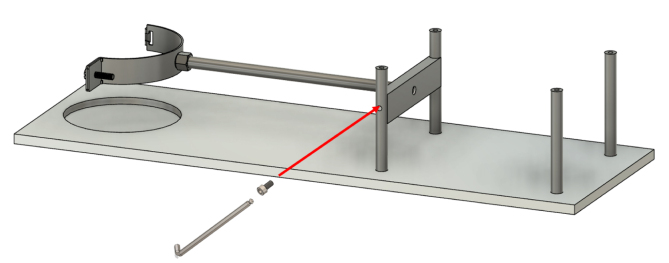
.

**Fig. 10:**
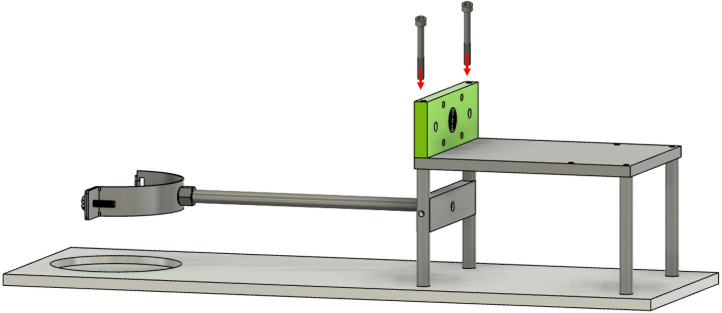
Front lead screw bracket installation.

The front lead screw bracket is mounted on the supporting frame using two M6x70 screws, as shown in Fig. 10. The bracket features a ball bearing positioned in its designated groove and four linear bearings secured with grub screws (Fig. 10).

**Fig. 11:**
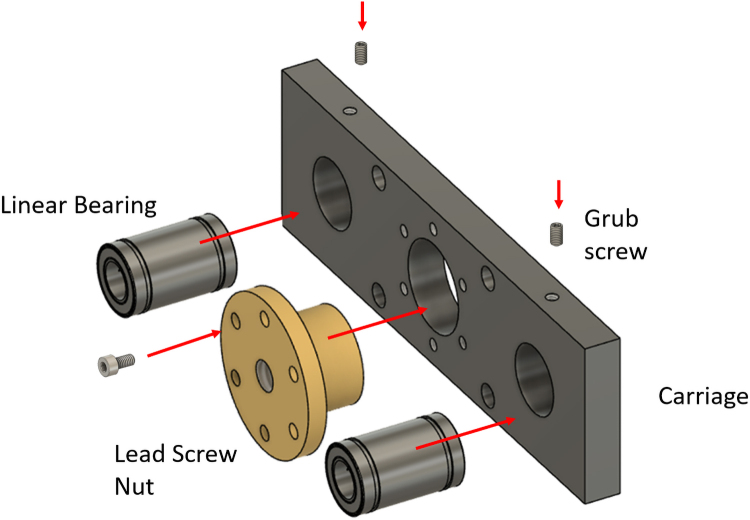
Preparation of the carriage.

The lead screw nut and the two linear bearings are installed on the carriage as shown in Fig. 11.

**Fig. 12:**
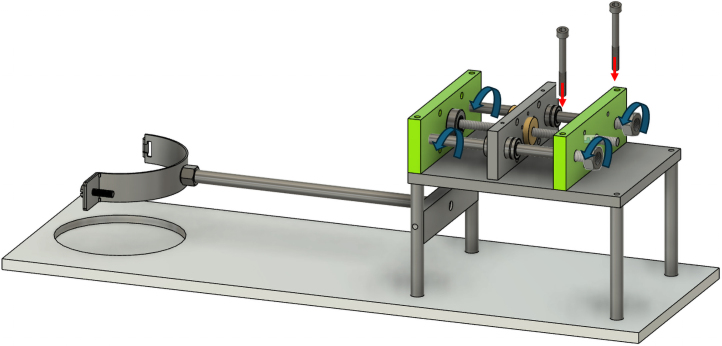
Assembly of the lead screw and back lead screw bracket onto the supporting frame.

At this point, it is possible to insert the lead screw into the lead screw nut. Next, two clamping shaft collars are installed: one at the front end of the lead screw and the other on the opposite side of the carriage. The back lead screw bracket is then positioned by sliding the lead screw through it. This assembly is placed onto the supporting frame, with the two linear guide rods passing through the linear bearings of the carriage. The front end of the lead screw is screwed into the front lead screw bracket, while the back end is secured with a nut, as illustrated in Fig. 12.

**Fig. 13:**
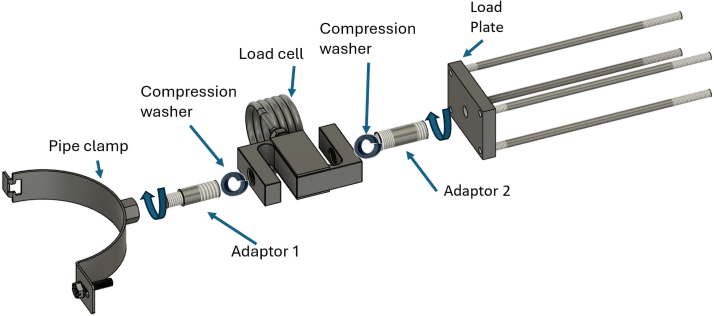
Assembly of the sliding arm.

To assemble the sliding arm, begin by attaching the pipe clamp (M10) to the load cell (M12) using a bespoke adaptor (Adaptor 1, M10 to M12) and a compression washer. Then, connect the opposite end of the load cell to the load plate (M10) using Adaptor 2 (M12 to M10) and another compression washer. The load plate is connected to the carriage of the linear motion system via four support rods, ensuring proper alignment and mechanical stability during loading. Fig. 13 illustrates the complete assembly of the sliding arm.

**Fig. 14:**
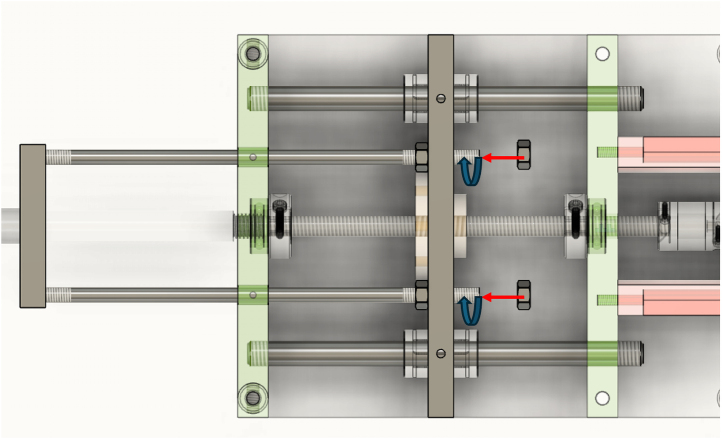
Attachment of sliding arm to the carriage.

The four carriage rods are first inserted through the linear bearings mounted on the front bracket of the device. An M6 nut is then threaded onto each rod before passing the rod through its corresponding hole in the carriage (pushing plate). Once the rods are in place, a second M6 nut is added to each rod on the opposite side of the carriage. The two nuts on each rod are then tightened against the carriage, effectively clamping it between them and ensuring a stable and rigid connection. Fig. 14 illustrates this assembly step.

At this stage, the motor is installed to complete the drive system. A bellows coupling was selected to connect the lead screw to the stepper motor shaft due to its flexibility and ability to compensate for minor misalignments. This type of coupling offers several advantages, including high torsional stiffness for efficient torque transmission, backlash-free operation, and vibration absorption to reduce wear on motor and screw components.

**Fig. 15:**
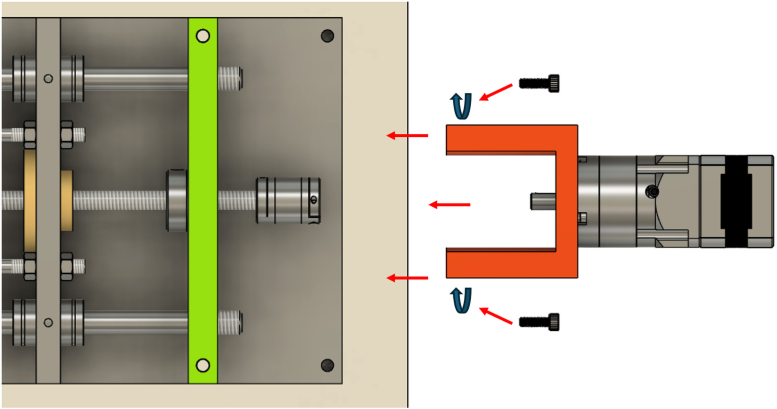
Installation of the stepper motor.

The coupling is first secured to the end of the lead screw. Separately, the stepper motor is mounted onto the gearbox, and this motor–gearbox assembly is fixed to a dedicated motor bracket. Next, the gearbox shaft is inserted into the free end of the coupling and fastened in place. Finally, the motor bracket is attached to the rear bracket of the RSSA frame, aligning the motor shaft with the lead screw to ensure proper fit and function. Fig. 15 illustrates this final connection.

Once the stepper motor is mounted on the RSSA device, the setup is complete.

The stepper motor was selected to generate sufficient axial force to shear the soil reinforced by plant roots. Based on the literature review, the maximum shear stress due to root reinforcement was estimated to be approximately 30 kPa [11], [28], only the self-weight is applied as the vertical force. This value was then used to determine the maximum linear force, F (N), required from the motor, calculated as:

(1) $$\tau =\frac{F}{A}\to F\left(\text{kN}\right)=\text{30 000}\;\text{Pa}\cdot \text{0.0079}\;\text{m}{}^{2}=\text{237}\;\text{N}$$

where A (m^2^) represents the shear area, corresponding to the inner diameter of the pipe, and τ (Pa) the shear stress.

Eq. (1) was also used to select the appropriate load cell for mounting on the RSSA device.

The power screw torque-force relationship used to convert this force into torque for motor selection is given by Budynas et al. [29]:

(2) $$T_{R}=\frac{F\;d_{m}}{2}\;\left(\frac{l+\pi \;f\;d_{m}\;\sec\alpha }{\pi \;d_{m}-f\;l\;\sec\alpha }\right)$$

where F (N) is the axial load, $d_{m}$ (m) is the pitch diameter, l (m) is the lead (i.e. the distance the nut moves parallel to the screw axis when the nut is given one turn), α (°) is the lead angle, f (-) is friction factor (≈0.15 [29]), and $T_{R}$ (N m) is the torque required to overcome the thread friction and drive the load. An additional torque component due to the collar bearings should be included in Eq. (2). It is considered good practice to incorporate collar bearings between the rotating and stationary components when a power screw is subjected to an axial load. This additional contribution is calculated as:

(3) $$T_{collar}=\frac{F\;f_{c}\;d_{c}}{2}$$

where $f_{c}$ (-) is the coefficient of collar friction (≈0.17 [29]), and $d_{c}$ (m) is the mean collar diameter.

Table 3 summarizes the values used to calculate the torque in Eqs. (2), (3).

The maximum torque is calculated by summing the value obtained from Eq. (2) and twice the torque from Eq. (3), as two collars are used, resulting in 0.86 N m. Therefore, a NEMA 17 stepper motor (maximum torque of 0.44 N m) with a 10:1 gearbox ratio was selected. With a 10:1 gearbox, the system achieves a torque amplification, allowing for a maximum shear stress of approximately 150 kPa to be applied during testing.

**Table 3:** Input parameters for torque estimation.

Variable	Definition	Value
d	Pot diameter	0.1 m
A	Shear area	7.9 × 10^-3^ m^2^
F	Axial force	237 N
$d_{m}$	Pitch diameter	10 × 10^-3^ m
l	Lead	2 × 10^-3^ m
α	Thread angle	15°
f	Friction factor	0.15
$f_{c}$	Collar friction factor	0.17
$d_{c}$	Collar diameter	15 × 10^-3^ m

## 6 Operation instructions

**Fig. 16:**
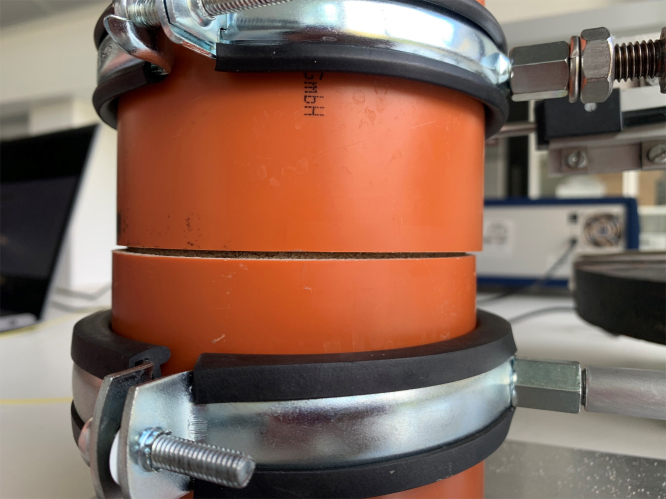
Close-up image of the clearance between the upper and lower PVC pipe sections prior to testing.

The pot containing the plant, ready for testing, is placed onto the shear device, see Fig. 17. The groove on the base plate of the device ensures that the pot is positioned correctly (Fig. 17(a)). Once in place, the two pipe clamps are tightened to securely hold the pot, see Fig. 17(b). At this stage, the tape holding the two halves of the pot together can be removed. After removing the tape joining the two pipe halves, care is taken to ensure a small clearance (approximately 1 mm) between the upper and lower PVC sections, so that they do not come into contact during shearing (Fig. 16).

The horizontal displacement transducer should then be adjusted to ensure it makes proper contact with the moving half of the pot. Once these steps are completed, the shear test can be performed.

**Fig. 18:**
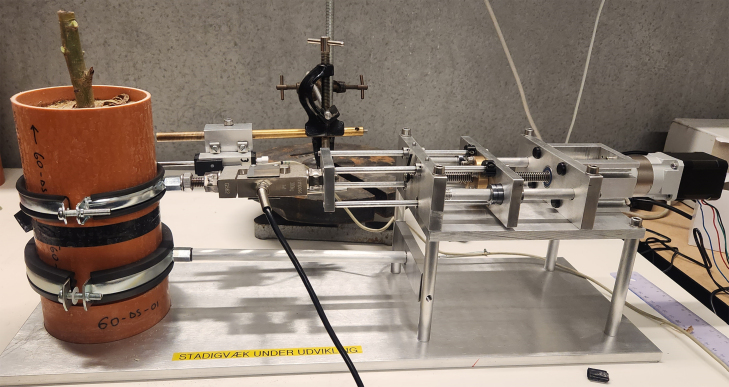
The RSSA device setup with a sample installed and sealed with tape along the mid-section during preparation. The tape is removed immediately before testing to expose the shear plane.

Fig. 18 shows the complete setup, including the sample in place, the stepper motor, and the sensors, all properly aligned and prepared for the shear test.Fig. 17Placement and securing of the pot on the RSSA device prior to the shear test: (a) placement of the pot; (b) securing the pot on the shear device.

**(a):**
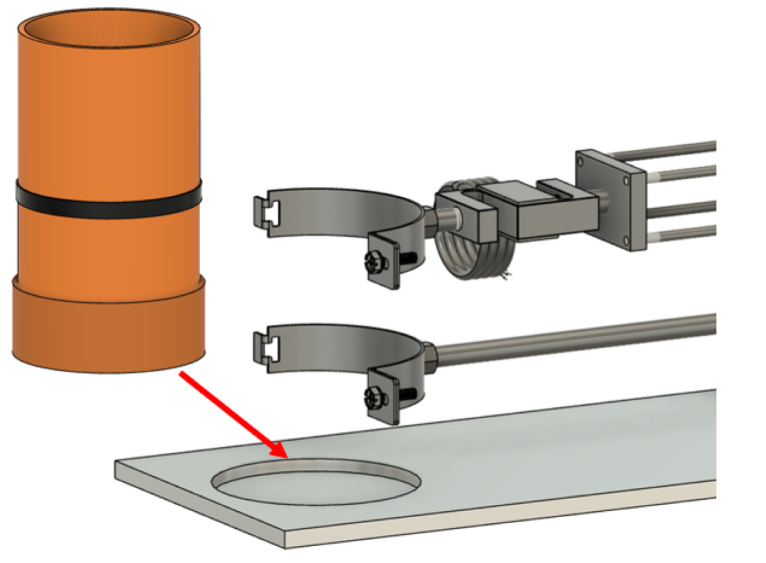
.

**(b):**
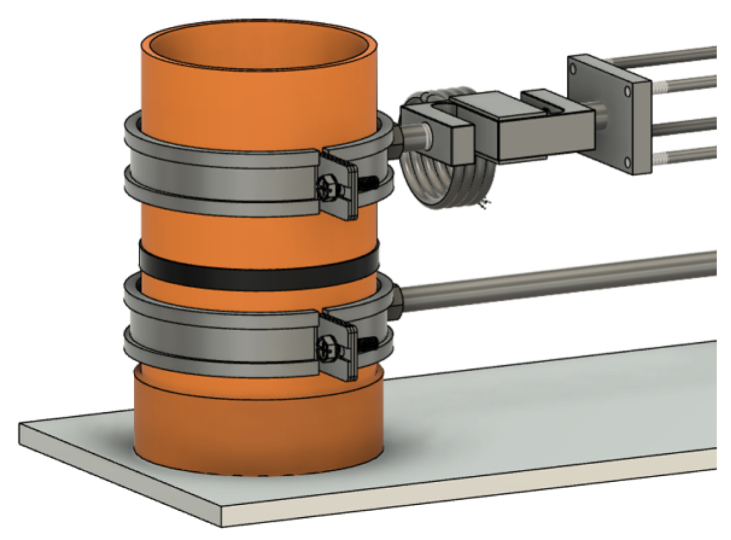
.

## 7 Validation and characterization

The shear test device was validated through experiments to ensure its reliability in assessing the influence of plant roots on soil shear strength. The validation process involved conducting a total of five tests. All tests were performed under self-weight, with no externally applied normal load. For comparative testing, the intended protocol was to pair rooted and unrooted specimens at the same water content so that differences in response reflect root reinforcement (soil + suction + roots vs. soil + suction). In this validation study, samples were tested at a water content of approximately 20%, to demonstrate device operation.

Four tests were performed on a sample consisting only of sand with a water content of approximately 20%, a condition chosen to ensure reliable and consistent measurements from the device. Two of these tests were conducted using the Arduino-based setup, while the other two were performed with the Raspberry Pi-based setup to compare their performance. Additionally, one test was carried out using a rooted-soil sample with the Arduino-based system. This test served as a proof of concept, demonstrating the feasibility of testing plant-root interactions.

**Fig. 19:**
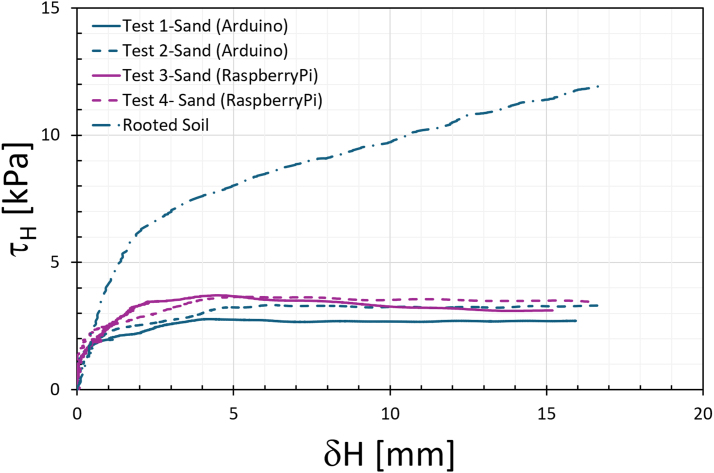
Comparison of direct shear test results for sand and rooted sand.

Fig. 19 presents the results of the five validation tests, showing the typical trend seen in direct shear tests on soil. The behavior observed in the direct shear tests aligns with documented trends in geotechnical literature. For loose sand shear stress increases gradually till it reaches a constant value [30], [31]. In the four tests conducted on sand with 20% water content, the shear strength at 15% horizontal deformation (equivalent to 15 mm of displacement on a 100 mm diameter sample) was measured at 3.0(5) kPa.

When testing the rooted soil sample, the shear strength increased significantly to approximately 12 kPa. Unlike the sand-only samples, the rooted sample exhibited continuous strain hardening, with shear strength increasing progressively throughout the test. This behavior suggests that the presence of roots enhances resistance to shearing and improves the material’s ductility. These observations are consistent with the findings of [11], who also reported increased shear strength and greater deformation capacity in root-reinforced soils.

**Fig. 20:**
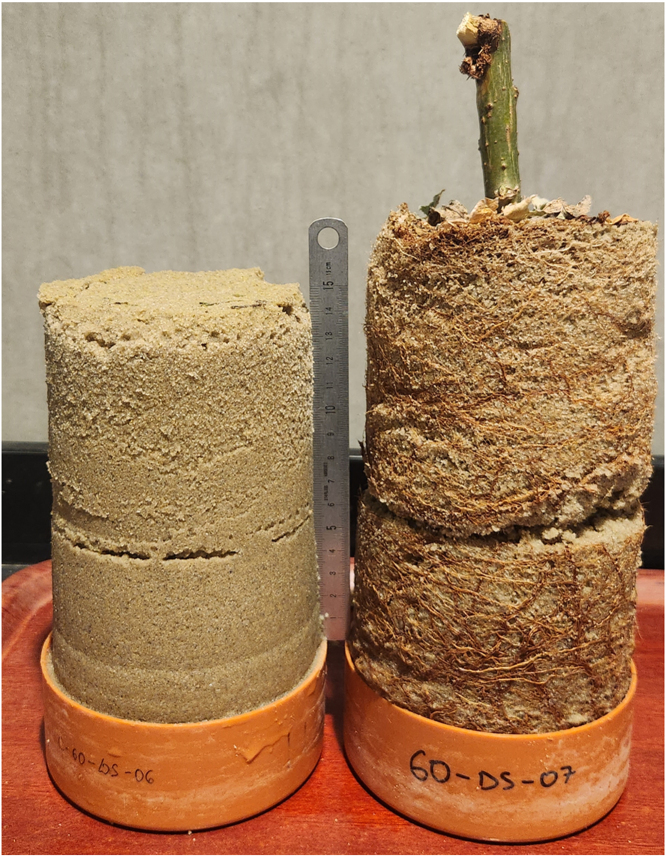
Comparison of the sheared samples after testing. On the left, the sand sample; on the right, the rooted soil sample. The apparent difference in height is due to sample disturbance during the removal of the PVC pipe from the rooted sample after the test.

Fig. 20 shows the shear plane clearly visible after removing the PVC pipe, highlighting that the shear failure occurred along the intended plane in both samples.

During shearing, material loss from the exposed shear surface was not observed, likely due to the partial saturation of the soil and the resulting suction, which maintained sample integrity. Photographs in Fig. 21 show the samples during shearing for both sand (Fig. 21(a)) and rooted sand tests (Fig. 21(b)), confirming that no material was lost as the top box displaced. A side view of the device during shearing is provided in Fig. 21(c). To further minimize the risk of soil dropping during large displacements or tests on drier samples, a removable collar can be attached around the upper edge of the lower pipe half, as illustrated in Fig. 21(d).

In this validation study, the imposed displacement was limited to approximately 18 mm, as the primary objective was to confirm the functionality of the apparatus rather than to fully characterize the strength of rooted soil. Although the device can achieve up to 50 mm of horizontal travel, the present tests were constrained by the available LVDT sensor (25 mm range) and were stopped once the setup had been successfully validated. Within this deformation range, the rooted sample exhibited continuous strain hardening, without reaching a plateau in shear strength. This response is consistent with previous studies, where root-reinforced soils often show increasing resistance with deformation and no distinct peak depending on root density and interlocking mechanisms [11], [17], [32], [33], [34]. Capturing a plateau or peak strength will require sensors with greater displacement capacity to fully mobilize shear resistance. This will be a focus of future experimental campaigns.Fig. 21Photographs illustrating the behavior of the samples during shearing and additional configurations of the RSSA: (a) sand sample during shearing; (b) rooted sand sample during shearing; (c) side view of the device under shear displacement; (d) scheme of the RSSA assembly showing the location of a possible retaining plate at the shear plane.

**(a):**
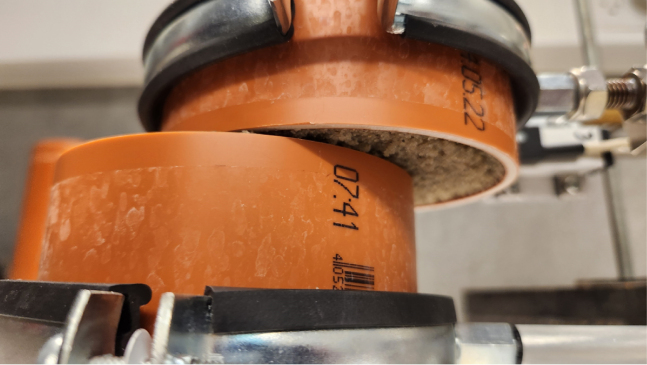
.

**(b):**
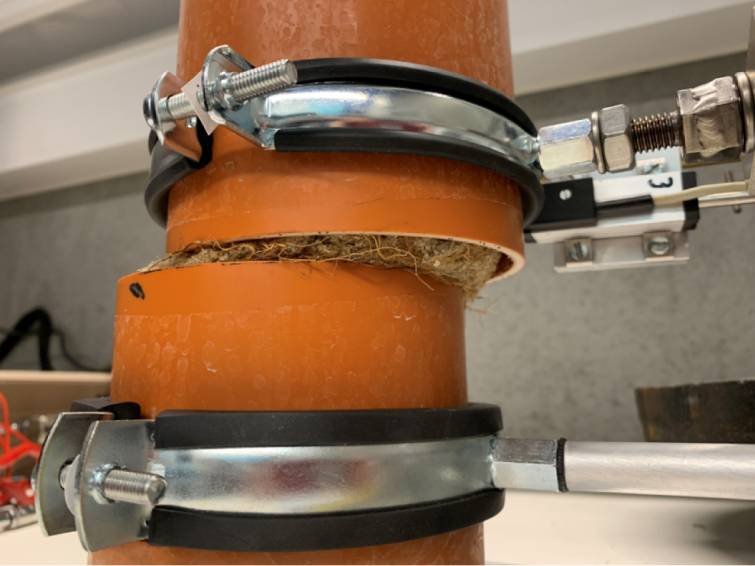
.

**(c):**
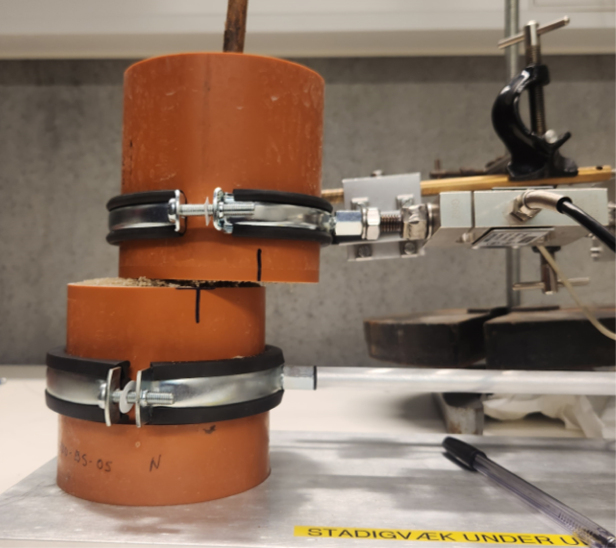
.

**(d):**
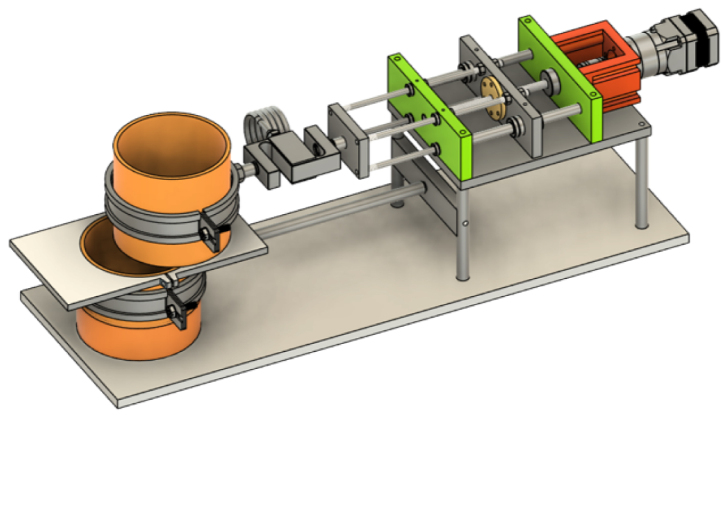
.

### 7.1 Limitations and future upgrades

The current RSSA operates under self-weight only, and Mohr–Coulomb parameters (cohesion c and friction angle ϕ) cannot be obtained without external normal stress. A simple way to extend the design is to add a dead-weight module. One option is a rigid loading cap placed on the specimen and connected to a plunger that carries the weights. To prevent eccentric loading when the upper half moves, the plunger can be guided laterally by a small carriage running on a horizontal linear rail. The carriage only provides side guidance and clearance is left so that all vertical load is transferred directly through the plunger to the specimen, not into the rail. A spherical seat or low-friction puck between the plunger and loading cap can be used to reduce parasitic horizontal forces. For a 100 mm sample, normal stresses of 5 kPa, 15 kPa and 30 kPa correspond to about 4 kg, 12 kg and 24 kg of weights.

Another option is to seal the specimen by closing the slots between the lid and the pipe and adding a flexible membrane across the split at the shear plane, then connect the top cap to a vacuum or pressure line to impose a controlled pore-pressure boundary. This allows modulation of the stress state (i.e., net normal stress and/or matric suction), rather than applying an external total normal stress [35].

### 7.2 Conclusion

Understanding the impact of plant roots on soil strength is increasingly critical in geotechnical and environmental engineering. However, existing testing methods often rely on expensive, bulky, and highly specialized equipment, limiting their accessibility and reproducibility. To overcome these challenges, we developed a cost-effective, compact, and easily manufacturable alternative. By integrating Arduino or Raspberry Pi, our system not only simplifies operation and enhances data acquisition but also offers a fully open-source software framework. This allows users to perform routine tests with minimal setup while enabling advanced users to modify and expand the system for more complex testing requirements. Unlike traditional setups, our device allows for direct testing of rooted soil within the same pot used for plant growth, preserving undisturbed root-soil interactions. This innovative approach provides researchers and engineers with a practical, adaptable, and widely accessible tool to advance the study of root reinforcement in soil stability. At this stage, the apparatus supports comparative testing under self-weight only; concepts for applying controlled normal load are outlined as a future upgrade. In practice, the apparatus allows strength measurements of rooted soil at various water contents (soil+suction+roots) and direct comparison to barren soil specimens at matched water contents (soil+suction).

## CRediT authorship contribution statement

**Gianmario Sorrentino:** Writing – original draft, Validation, Software, Methodology, Investigation, Formal analysis, Conceptualization. **Gerald Innocent Otim:** Writing – review & editing, Validation. **Alena Zhelezova:** Writing – review & editing, Conceptualization. **Irene Rocchi:** Writing – review & editing, Project administration, Funding acquisition.

## Declaration of competing interest

The authors declare that they have no known competing financial interests or personal relationships that could have appeared to influence the work reported in this paper.
